# A revision and key to the genera of Afrotropical Mantispidae (Neuropterida, Neuroptera), with the description of a new genus

**DOI:** 10.3897/zookeys.184.2489

**Published:** 2012-04-21

**Authors:** Louwtjie P. Snyman, Michael Ohl, Mervyn W. Mansell, Clarke H. Scholtz

**Affiliations:** 1 Department of Zoology and Entomology, University of Pretoria, Pretoria 0002, South Africa; 2 Museum für Naturkunde Berlin, Invalidenstr. 43, 10115 Berlin, Germany

**Keywords:** Mantispidae, Neuroptera, Afrotropical, lacewings, key

## Abstract

The Afrotropical Mantispidae genera have previously been neglected and are poorly known. The genera are revised and redescribed. A new genus *Afromantispa* Snyman and Ohl is described with *Afromantispa tenella* **comb. n.**as type species. *Perlamantispa* (Handschin, 1960) is synonymised with *Sagittalata* Handschin, 1959. The new combinations within the genus include *Sagittalata austroafrica* **comb. n.**, *Sagittalata bequaerti* **comb. n.**, *Sagittalata dorsalis* **comb. n.**, *Sagittalata girardi* **comb. n.**, *Sagittalata nubila* **comb. n.,** *Sagittalata perla* **comb. n.,** *Sagittalata pusilla* **comb. n.**, *Sagittalata similata* **comb. n.**, *Sagittalata royi* **comb. n.**, *Sagittalata tincta* **comb. n. and** *Sagittalata vassei* **comb. n.** An illustrated key to the genera *Afromantispa* **gen. n.**, *Sagittalata* Handschin, 1959, *Mantispa* Illiger, 1798, *Cercomantispa* Handschin, 1959, *Rectinerva* Handschin, 1959, *Nampista* Navás, 1914, and *Pseudoclimaciella* Handschin, 1960 is provided. The wing venation of Mantispidae is redescribed. Similarities between the genera are discussed. Subsequent studies will focus on revising the taxonomic status of species, which are not dealt with in this study.

## Introduction

The superorder Neuropterida is considered to comprise a diversity of clades, many of which are characterized by a large number of plesiomorphic characters. It includes the orders Raphidioptera, Megaloptera and Neuroptera (Aspöck 2001; Aspöck et al. 2001; Winterton et al. 2010). The latter two are well represented in the Afrotropical Region, with approximately 1200 species in 15 families (Mansell 2010).

The significance of the order Neuroptera is well documented in fields other than taxonomy. All larvae are obligate predators, while adults are predacious or pollen-feeders and consequently fulfil vital roles in the functioning of natural ecosystems. The order is therefore ideal for studies in biological fields other than taxonomy (Picker 1984; Mansell 2002; Ohl 2004). Furthermore, their diverse range of habitats and life strategies make them ideal indicator species for global warming, fresh water health, and habitat fragmentation and destruction (Picker 1984; Mansell 2002; Ohl 2004). Despite their importance, some families are poorly studied with only a few groups having received any modern scientific scrutiny.

The Afrotropical Mantispidae are certainly one of the families that is in urgent need of revision (Ohl 2004, 2005). Reasons for this include the taxonomic complexity of the family and the confusing scientific legacy left by most previous authors. Adults are morphologically difficult to distinguish and the lack of comprehensive modern revisions makes the taxonomic status of most taxa difficult to interpret. They are consequently not easily accessible to other non-taxonomic research fields because of the paucity of taxonomic revisions or morphological keys to provide easy access to the group. Modern revisions and morphological keys are consequently of extreme importance (Ohl 2004, 2005).

The Mantispinae are the only subfamily known from the Afrotropical Region, and since the Afrotropical Mantispidae have received little attention, their potential impact on other fields of biology cannot be readily assessed. The positive impact of other families of Neuroptera has proven to be of great value in fields such as agriculture as biological control agents taxonomy (Picker 1984; Stelzl and Devetak 1999; Mansell 2002). Furthermore, revisions are important for biodiversity inventories, not only for the relevant country, but also for global biodiversity (Mansell 2002; Ohl 2004, 2005).

This study forms part of two programmes, a southern African initiative and a global programme. The first will form part of the programme: ‘Monitoring lacewings (Insecta: Neuroptera) in southern Africa’ (Mansell 2002). This initiative has five main areas of focus or operational components, namely (1) Biodiversity audit, (2) Systematic revisions and phylogenetic analysis (3) Larval biology and ecological requirements (4) Distribution patterns and predictive modelling (5) Conservation status and protective measures, which are all underpinned by a relational database of Afrotropical Neuroptera (Mansell and Kenyon 2002). The second programme entitled, ‘Towards a global inventory of Mantispidae - the state-of-the-art in mantispid taxonomy’ has revisions and morphological key generation as the central theme (Ohl 2004, 2005). The current study thus contributes significantly to improving both our local and international knowledge of this poorly known group.

The ultimate long term aim of the study is to resolve the taxonomy of Afrotropical Mantispidae in order to facilitate further research on the group. Since the Afrotropical Mantispids has never received a comparative and large scale revision, this manuscript will form the basis of long term directional research. To achieve this, generic groups was redefined on the basis of clear autapomorphies to support their monophyly, and future molecular studies will be carried out to determine whether these morphologically-defined genera are supported by DNA evidence. Current as well as subsequent studies on the taxonomy and biology of the species can be carried out within the context of these generic concepts.

## Biology of Mantispinae

Female Mantispinae lay large batches of stalked eggs ranging from several hundred to several thousand (Redborg and MacLeod 1985; Redborg 1998). The substrate to which the eggs are attached varies and oviposition preference behaviour of females has not been investigated (Hoffman 1992; Redborg 1998). It has however been hypothesised that females do select specific areas, *e.g*. those with high spider prevalence. Some support for this observation has been found, but has not been tested. Stalked eggs are common in Neuroptera and it has been hypothesised that this adaptation is due to the cannibalistic behaviour of the first instar larvae (Penny 1982). Mantispine larvae, however, are not generalist predators and all known species are predators of spider eggs and have been reported to only kill fellow larvae once inside an egg-sac (Redborg and MacLeod 1985; Hoffman 1992; Redborg 1998). The reason for the evolution of stalked eggs, consequently, remains unclear.

A campodeiform, triungulin larvae hatches from the stalked eggs and uses one of two strategies for locating a food source (Hoffman and Brushwein 1990, 1992; Hoffman 1992; Redborg 1998). Either the larvae, termed obligate egg-sac penetrators, actively move about in search of an egg-sac or those termed obligate spider boarders will board a spider (Hoffman and Brushwein 1990, 1992; Hoffman 1992; Redborg 1998). It is thought that larvae that use the former strategy are attracted to spider silk (Redborg and MacLeod 1985). The second strategy includes phoretic behaviour. The larva is equipped with caudal suckers that are attached to a substrate. The head and thorax are then raised upwards to assume the phoretic position (Redborg and MacLeod 1985; Redborg 1998). The larva will sway back and forth with legs outstretched waiting for a spider to pass by. Once a spider has been boarded, the larva will usually wrap itself around the pedicel of the spider or it enters a book lung (Redborg and MacLeod 1985; Redborg 1998). A first instar larvae attached to the pedicel of an immature Clubionoidea spider has been recorded from Eocene Baltic amber (Ohl 2011). Some species are known to make use of both strategies and this is termed facultative boarders/penetrators (Redborg and MacLeod 1985; Redborg 1998). Once inside an egg-sac, the larva produces an allomone that limits the development of the spider eggs to increase the feeding time of the larva (Redborg 1983).

The larvae undergo a unique ontological pattern. It has been proposed that the developmental pattern can be termed hypermetamorphic ontogeny but some authors do not agree (Brauer 1869; Redborg 1998). The development is certainly not as complex as that of beetles in the family Meloidae, but much more complex than most known insect ontogenies (Redborg 1998). The first-instar moults into a less mobile, less sclerotised second-instar. The second-instar is eruciform to scarabaeifrom. The final- and third-instar is grub-like, immobile and scarabaeiform (Redborg 1998). As in other Neuroptera larvae, the Malpighian tubules are modified into silk-producing glands that are used to produce a cocoon in the now-empty egg-sac (Bissett and Moran 1967). When the construction of the cocoon is initiated, remaining eggs are ignored. However, if the entire batch of spider eggs is consumed spinning is initiated. It has been found that there is a direct relationship between the adult and the amount of food ingested by the third instar larva, leading to extreme variation in adult size (Redborg and MacLeod 1985). Redborg and MacLeod (1985) found individuals twice the size of other individuals of the same species that could be correlated with the number of eggs consumed. The adults are generally predacious, but it has been suggested that some may be pollinators (Tjeder 1963; Hoffman 1992). The evidence is not conclusive and flowers might just be a suitable platform for ambushing prey as is manifest by some praying mantids (Mantodea) and crab spiders (Thomisidae).

The mating behaviour of mantispids has received some attention (Eltringham 1932; Redborg and MacLeod 1985; Redborg 1998). The adults face each other and a series of movements are followed, probably for recognition purposes since cannibalism is common when adults encounter one another (Eltringham 1932; Redborg and MacLeod 1985; Hoffman 1992; Redborg 1998). An organ termed Eltringham’s extrusible gland (EEG) is situated dorsally in the fourth and fifth abdominal segments of some male mantispids (Eltringham 1932). It is thought that the gland secretes a pheromone that has a calming effect on the female (Eltringham 1932; Redborg and MacLeod 1985; Hoffman 1992; Redborg 1998). The pheromone is probably distributed by short and regular bursts of ‘wing flapping’ during the mating ritual (Redborg and MacLeod 1985; Hoffman 1992). Mating may take more than 24 hours and the whitish spermatophore can be seen after mating while entering the female genitalia (Redborg and MacLeod 1985).

## Systematic status of Afrotropical Mantispidae

### Taxonomic complexity

The Afrotropical Mantispidae are taxonomically extremely complex. Interspecific morphology differs only slightly, while marked intraspecific variation adds to the complexity. Appropriate distinguishing characters must consequently be carefully sought. The literature on southern African taxa is limited. Most of the species descriptions are vague and not intelligible, often lacking sufficient illustrations. This is probably because several authors were not Neuropterists and described the specimens using inappropriate characters and often overlooked important distinguishing criteria. As an example, Mansell (2010) found that Longinos Navás described over 764 Afrotropical neuropteran taxa (mostly species) of which 50% have already proven to be invalid. Museum material reveals that Navás misspelled names and misidentified several species, including type specimens, and re-described some species that he had originally described himself, often classifying the re-described species into a different genus. All Navás’ descriptions and names consequently require careful evaluation.

### Previous works

Previous authors did little comparative studies and described new taxa without conclusively capering them with similar taxa (Handschin 1959, 1960a, b, 1963). Therefore, the main taxonomic impediment in the family appears to be synonymy. No complete, comparative revisions of the generic groups has previously been published, with only Poivre (1982b) appempting to compare some of the genera by using a matrix consisting of six relatively weak characters. To use the matrix for identification purposes, the specimen must be dissected and consequently destroyed. Furthermore, the matrix proved to be inadequate to identify several specimens to generic level.

The most recent publications on the Afrotropical taxa were by Poivre in the early 1980’s (Poivre 1980; 1981a, b; 1982a, b; 1984a; 1985). Several new species were described and a limited revision was done on some of the West African species. Poivre’s publications are complex and his statements difficult to interpret. He frequently used anatomical morphological characters such as internal genitalic stuctures for descriptions, but did not provide comparative descriptions or keys. Several of the Poivre types (*e.g*. *Pseudoclimaciella ivoiriensis* Poivre, 1982b and *Pseudoclimaciella cachani* Poivre, 1982b) housed in MNHN are completely dissected, macerated and mounted on slides. The major concern is the loss of information from material preserved on slides. Colours and patterns, which are of extreme importance, are both difficult to communicate in articles and are lost when using slides as a method of preservation.

### Nampista Navás, 1914

This Palaearctic genus was recently revised by Ohl (2009) so does not require redescription. It is incorporated into the key because *Nampista auriventris* (Guérin-Méneville), *Nampista africana* (Esben-Petersen) and *Nampista ragazziana* (Navás) also occur in African countries (Egypt, Sudan, Eritrea, Ethiopia, Djibouti and possibly Somalia) bordering the Arabian Peninsula.

### Mantispa

*Mantispa* Illiger, 1798 is treated as a Palaearctic genus and is not redescribed in this study. Only a few specimens were available for this revision. Material is currently being obtained for a full comparative study of *Mantispa* and *Sagittalata* Handschin, 1959.

### Mantispa and Perlamantispa

It has been suggested that *Perlamantispa* Handschin, 1960 may be a synonym of *Mantispa* (Aspöck et al. 1980a). For the purposes of this study, the genera have been kept separate (see discussion below).

## Material and methods

**Figure 1.**
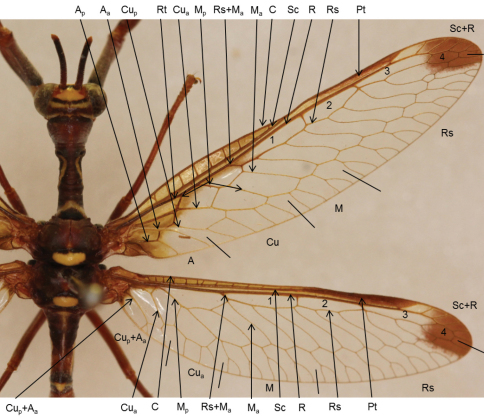
Wing venation of Mantispidae. **A** Anal **C** Costa **Cu** Cubitus **M** Median **Pt** Pterostigma **R** Radius, Radial cells 1, 2, 3 and 4 **Rt** Radial triangle **Rs** Radial sector **Sc** Subcosta **+** indicates fused veins **_p_** posterior **_a_** anterior.

***Wing terminology*:** This study followed the wing terminology of Lambkin (1986a, b) with additions from Ferris (1940), Aspöck et al. (1980b) and Hoffman (1992). Some minor changes to vein systems at the wing margins have been made. The changes include a different interpretation of Sc+R, Rs, M, Cu and A (anal vein system in only the hind wing) (Fig. 1). The wing venation of Mantispidae is complex with controversy abounding. The complexity is due to the major veins that are often fused and the relatively reduced number of minor veins. The median fused with the radius in both the forewing (FW) and the hind wing (HW) have been interpreted differently by several authors (Ferris 1940; Aspöck et al. 1980b; Lambkin 1986a, b), the most recent being Machado and Rafael (2010).

In the FW Rs+M_a_ splits into Rs and M_a_ at the first fork (Fig. 1). The M_a_ vein follows a path directly to the posterior margin. The Rs form the three radial cells and the anterior half of a hexagon-shaped cell (radial cell 4) before reaching the apical margin. In the FW Ferris (1940) interpreted the M_a_ as a vein fused with Rs that separated from the Rs by forking into yet another anterior and posterior M_a_ with the anterior M_a_ following the fusion with the radial sector up to the first crossvein originating from the first radial cell. This interpretation (or assumption as referred to by Ferris) has been widely adopted with only Lambkin questioning the statement by explaining several different scenarios (Lambkin 1986a). In the present study only the proximal border of the first radial cell is considered to be a fusion between M_a_ and Rs. Consequently, no crossvein originating from a radial cell is considered to be fused with M_a_. The interpretations of the mantispid wing are illustrated in Figure 1.

***Male genitalia*:** No dissections were made because it proved to be unnecessary for the delimitation of monophyletic groups. Preliminary studies indicated that internal genitalic structures might not be a necessary character for the elucidation of the Afrotropical Mantispidae genera even though it is valuable for species delimitation. The only genus where the ectoprocts as well as the pseudopenis are autapomorphic for the Afrotropical genera is *Cercomantispa* Handschin, 1959 (Fig. 4f; also see discussion below *Cercomantispa* and *Rectinerva* Handschin, 1959). Tjeder (1963) noted that the pseudopenis is not used for sperm transferal and thus wrongly termed in previous work (Flagellum in Ferris 1940; penis/spinasternum in Handschin (1959); spinasternum in Poivre 1980; 1981a, b; 1982a, b; 1984a; 1985). In all other groups, such as *Pseudoclimaciella* Handschin 1960 there is too much genitalic variation whereas other characters proved consistent. Eltringham’s extrusible gland (EEG) is visible between the fourth and fifth abdominal tergite of some males without dissection and it was noted when the gland is present.

***Studied genera*:** Since the genera studied here are all distributed in the Afrotropics, the autapomorphies have not been compared to all Mantispidae genera. The genus *Mantispa* Illiger, 1798 is not assigned an autapomorphy because of the confusion between this genus and *Sagittalata* Handschin, 1959 (see *Sagittalata* below) that resulted in the lack of a character rather than the presence of one.

***Madagascar*:** The Madagascar fauna was revised by Handschin (1963). The two genera occurring on Madagascar are not included in this revision. The type specimens of both *Paulianella* Handschin 1960a and *Madantispa* Fraser, 1952 should be in MNHN but have apparently been lost. Due to the lack of identified specimens of either of the genera in museum collections, as well as the inadequate descriptions, they are excluded from this revision. The fauna of Madagascar will only be revised once the type specimens are either located or sufficient specimens are collected.

Name combinations and museums holding valuable collections were identified using LDL (Lacewing Digital Library) and the Ohl catalogue (2004). All specimens studied were dried pinned specimens. The collections studied are housed in the following institutions:

**SANC** South African National Collection of Insects, Pretoria, South Africa

**MZB** Museo de Zoologia, Barcelona, Spain

**MRAC** Musée Royal de l’Afrique Centrale, Tervuren, Belgium

**ZMB** Museum für Naturkunde an der Humboldt-Universität, Berlin, Germany

**MNHN** Museum National d’Histoire Naturelle, Paris, France

**DMNH** Ditsong Museum of Natural History (formerly Transvaal Museum of Natural History), Pretoria, South Africa

### Genus Afromantispa

Snyman & Ohl gen. n.

urn:lsid:zoobank.org:act:23AFF0D1-4D05-4E15-8B91-79F32AE4A9D0

http://species-id.net/wiki/Afromantispa

Afromantispa Snyman and Ohl gen. n. Type species: *Afromantispa tenella* (Erichson, 1839: 169) comb. n., designated here.

#### Remarks.

Both Handschin (1959, 1960a) and Poivre (1980, 1982b) considered the Afrotropical Mantispidae to be part of *Mantispa* without comparing the Afrotropical species to the type species of *Mantispa* (*Mantis pagana* Fabricius, 1775 (=*Mantispa styriaca* Poda, 1761)). The new genus *Afromantispa* can easily be identified and clearly differs morphologically from the Palaearctic *Mantispa* although the two genera are possibly closely related. (see discussion below). Species complexes within this genus must be investigated, with at least one species with a distribution from South Africa to the Arabian Peninsula lacking morphological variation. Sexual dimorphism has not been formally investigated, but it appears to be absent in the genus.

#### Distribution.

Widespread throughout Africa. A few species have been collected in the Palaearctic Region sharing borders with Africa such as Spain and the Arabian Peninsula.

#### Diagnosis.

**Figure 2.**
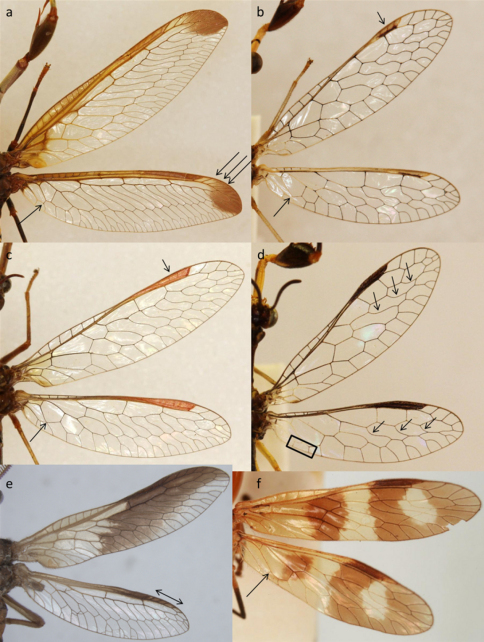
Wing variation in the Afrotropical Mantispinae. **a** *Pseudoclimaciella apicipennis* (Kolbe) **b** *Afromantispa* sp. **c** *Sagittalata* sp. **d** *Cercomantispa* sp. **e** *Nampista ragazziana* (Navás) **f** *Rectinerva braconidiformis* Handschin.

**Figure 3.**
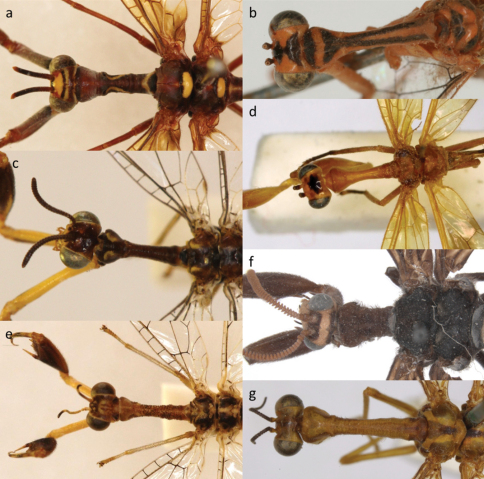
Pronotum variation in the Afrotropical Mantispinae **a** *Pseudoclimaciella apicipennis* (Kolbe) **b** *Sagittalata hilaris* (Navás) **c** *Cercomantispa* sp. **d** *Rectinerva braconidiformis* Handschin **e** *Afromantispa* sp. **f** *Nampista ragazziana* (Navás)**g** *Mantispa mandarina* Navás.

**Figure 4.**
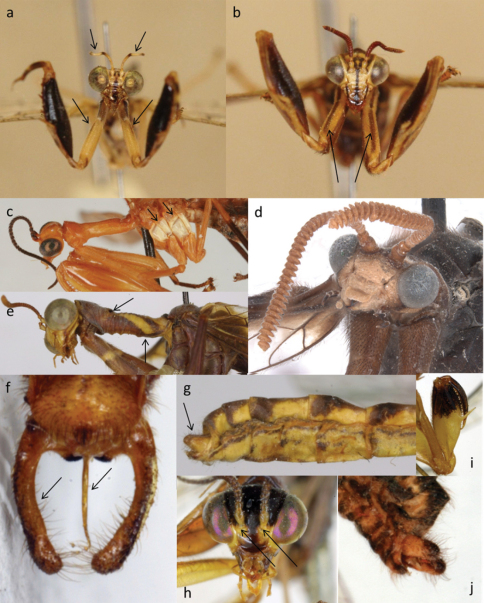
Apomorphic characters of the Afrotropical Mantispinae **a** *Afromantispa* sp., antennal band and discontinuous fore-coxal line **b** *Sagittalata* sp., continuous line on fore-coxae **c** *Rectinerva braconidiformis* Handschin, Elongated antennae and light pleura **d** *Nampista ragazziana* (Navás), lamellate flagella **e** *Pseudoclimaciella apicipennis* (Kolbe), black maculae and pronotal markings **f** Male *Cercomantispa perparva* (Esben-Peterson), elongated ectoprocts and pseudopenis **g**Male *Sagittalata* sp., slightly enlarged/swollen ectoprocts **h** *Cercomantispa* sp., anterior scape and pedicel always yellow **i** Male *Cercomantispa perparva* (Esben-Peterson), inner femoral surface.

Prothorax granulated; granules dark (Fig. 3e). Antennae with distinct yellowish white band in the apical third (Fig. 4a). Even species with pale antennae have a few darker flagellomeres two-thirds apically from the base of the antennae and on the apex to form a yellowish-white band. The crossvein between Cu_a_ and Cu_p_+A_a_ in hind wing attenuated or absent (Fig. 2b). These characteristics combined are unique to this genus and can be used to distinguish *Afromantispa* species from all other genera.

#### Description.

*Head*: Antennae moniliform; colour variable but all with conspicuous yellowish-white band in apical third; scape and pedicel yellow. Posterior vertex concave except for slight convex elevation directly posterior to and between antennal bases; median tubercle projection at posterior margin of vertex, vertex not visible in lateral view. Compound eyes large, each eye slightly broader medially at epistomal suture. Labrum circular. Mandible with dark apices; inner margins dark.

*Thorax*: Pronotum narrow and elongated; prothorax longer than pterothorax; granulated; granules dark; pronotum transversely slightly wrinkled or rugulose; setae present. Maculae slightly raised and inconspicuous; not pigmented in lighter coloured species, pigmented and shiny in darker species. Prozona slightly broader than base. Meso- and metathorax of similar size and distinctly separated by a deep cleft.

*Wings* (Fig. 2b): Wings always hyaline, lacking pigment except for the pterostigma. Pterostigma slightly concave in dorsal view; semi-circular and truncate appearance; pterostigma of most species with reddish appearance. Radial cell 1 and 2 of similar size with radial cell 3 smaller and narrower; a single crossvein from third radial cell to anterior margin (C). *Hind wings*: Crossvein between Cu_a_ and Cu_p_+A_a_ attenuated or absent; Cu_a_ with sharp angle to and from attenuated crossvein to form inverted triangle.

*Legs*: Median line on the anterior surface of the forecoxae never continuous from thorax to femur (Fig. 4a). Mid- and hind legs differ considerably between species. Fore tarsal claw reduced to a single claw lacking arolium; Mid- and hind pretarsal claws pectinate (5 -6 teeth) with the middle tooth elongated giving the claw a sharp triangular appearance; arolium present on mid and hind tarsi.

*Abdomen/Genitalia*: Males with ectoprocts slightly enlarged (Fig. 4j). Pseudopenis visible in lateral and dorsal view. EEG present. No morphological significance regarding the female genitalia.

#### Etymology.

The new genus name is a combination of *Afro*- and *Mantispa*, which emphasises the African distribution of this *Mantispa*-like taxon.

#### Included species.

Besides the type species, 18 confirmed and 7 unconfirmed species names will be added in the future. These numbers are, however, certain to change. Synonyms need to be identified and new species described. A subsequent full revision of *Afromantispa* is currently in progress. The genus for the time being will therefore be based on the type species only.

### Genus Mantispa

Illiger

http://species-id.net/wiki/Mantispa

Mantispa Illiger in Kugelann 1798: 499. Type species: *Mantis pagana* Fabricius, 1775: 278 (=*Raphidia styriaca* Poda, 1761: 101), by monotypy.
Amycla Rafinesque, 1815: 118. Unjustified emendation of *Mantispa* Illiger in Kugelann 1798. *Amycla* was considered an emendation of *Mantispa* Illiger in Kugelann 1798, by Neave 1939, 1: 167.
Mantispilla Enderlein, 1910: 346 (as subgenus of *Mantispa* Illiger in Kugelann 1798). Type species: *Mantispa indica* Westwood, 1852: 268, by original designation. Synonymised with *Mantispa* by Penny 1982: 217.

#### Remarks.

The genus was described by Illiger (1798) in a single sentence more than two centuries ago with the European species *Mantis pagana* Fabricius, 1775 (= *Mantispa styriaca* (Poda, 1761)) as the type species. It became the best-known Mantispidae genus probably due to the age of the genus, and several species described worldwide were incorrectly assigned to the genus. It therefore became the most speciose genus in the family Mantispidae (Ohl 2004; Machado and Rafael 2010). More recently, Hoffman (2002) proposed that revisions should focus on elucidating the true taxonomy of *Mantispa*. Newly described genera in the New World replaced the universal *Mantispa* name that is now restricted to the Old world (Hoffman 2002; Machado and Rafael 2010). *Mantispa* might be confined to the Old World but is probably only represented by a few Palaearctic and Oriental species and not by species from the Afrotropics.

#### Distribution.

Palaearctic genus with some species records from the Afrotropical Region. These countries include Morocco and countries bordering the Arabian Peninsula.

#### Diagnosis.

Ectoprocts sligtly swollen/enlarged. Pterostigma elongated and dark red. Prothorax with setae and slightly transversely rugulose (Fig. 3g). Fore coxae lack continuous median line on anterior surface (Fig. 4b).

### Genus Sagittalata

Handschin

http://species-id.net/wiki/Sagittalata

Sagittalata Handschin, 1959: 215. Type species: *Mantispilla hilaris* Navás, 1925: 573 (as “*Sagittalata hilaris* (Navás 1924) [sic]”), by original designation
Perlamantispa Handschin, 1960a: 191. Type species: *Mantis perla* Pallas, 1772: 14 (as “*Mantispa perla*”), by original designation. syn. n.

#### New combinations

*austroafrica* (Poivre)

*Perlamantispa austroafrica* Poivre, 1984b: 642. syn. n.

*bequaerti* (Navás)

*Mantispilla bequaerti* Navás, 1932: 279. Synonymized with *Perlamantispa bequaerti* (Navás) by Handschin, 1960a: 197.

*Mantispilla bequaerti* var. *decolor* Navás, 1932: 280. Synonymized with *Perlamantispa bequaerti* (Navás) by Handschin, 1960a: 197.

*Mantispilla kibumbana* Navás, 1936c: 355.. Synonymized with *Perlamantispa bequaerti* (Navás) by Handschin, 1960a: 197.

*Perlamantispa bequaerti* (Navás). As a new combination by Handschin 1960a: 197.syn. n.

*dorsalis* (Erichson)

*Mantispa dorsalis* Erichson, 1839: 168.

*Mantispilla hemichroa* Navás, 1931: 129.

*Mantispilla hypophoea* Navás, 1932: 279.

*Perlamantispa dorsalis* (Erichson). As a new combination by Handschin 1960a: 196.syn. n.

*girardi* Poivre

*Perlamantispa girardi* Poivre, 1982a: 194.syn. n.

*nubila* (Stitz)

*Mantispilla nubila* Stitz, 1913: 15.

*Mantispa nubila* (Stitz, 1913) syn. n.

*perla* (Pallas)

*Mantis perla* Pallas, 1772: 14.

*Mantispa christiana* Charpentier, 1825: 93. Synonymized with *Mantispa perla* by Erichson 1839: 167.

*Mantispa flaveola* Erichson, 1839: 168.

*Mantispa victorii* Guérin-Méneville, 1844: 391. Synonymized with *Mantispa perla* by Hagen 1858: 128.

*Mantispa perla* var. *brunnea* Navás, 1906: 102.

*Perlamantispa perla* (Pallas, 1772). As a new combination by Handschin 1960a: 191.syn. n.

*pusilla* (Pallas)

*Mantis pusilla* Pallas, 1772: 15

*Mantis brevicornis* De Geer, 1778: 620, pl. 46, fig. 9–10. Synonymized with *Mantispa pusilla* by Burmeister 1839: 967.

*Perlamantispa pusilla* (Pallas, 1772) As a new combination by Handschin 1960a: 191.syn. n.

*similata* (Navás)

*Mantispilla similata* Navás, 1922: 396.

*Perlamantispa similata* (Navás, 1922). Listed as valid combination in Ohl (2004) and LDL. syn. n.

*royi* Poivre

*Perlamantispa royi* Poivre, 1982a: 191. syn. n.

*tincta* (Navás)

*Mantispilla tincta* Navás, 1929: 107

*Perlamantispa tincta* (Navás, 1929). As a new combination by Handschin 1960a: 200.syn. n.

*vassei* (Navás)

*Mantispa vassei* Navás, 1909: 474.

*Mantispa (Mantispilla) lineatifrons* Enderlein, 1910: 346. Synonymized with *Perlamantispa vassei* by Handschin, 1960a: 193.

*Mantispilla sankitana* Navás, 1922: 395. Synonymized with *Perlamantispa vassei* by Handschin, 1960a: 193.

*Mantispilla burgeoni* Navás, 1923: 77., Probable synonym of *Perlamantispa vassei* according to Handschin, 1960a: 193.

*Perlamantispa vassei* (Navás, 1909) As a new combination by Handschin, 1960a: 193. syn. n.

#### Remarks.

Handschin seemed to confuse the female *Cercomantispa* specimens and the genus he described as *Sagittalata*. In his revision (1960a) he mentioned that types of *Sagittalata tristis* and *Sagittalata tristella* are both female and that he is certain they are *Sagittalata* species. He mentions that the wing venation and prothorax corresponds with *Sagittalata*. However, the complete fusion between the Cu_a_ and Cu_p_+A_a_ veins in the hind wing to form a rectangle (Fig. 2d) occurs in *Sagittalata tristis* (= *Cercomantispa tristis*) and *Sagittalata tristella* (= *Cercomantispa tristella*) corresponds with *Cercomantispa* and not with the type species *Sagittalata hilaris* or any of the other species (*Sagittalata lugubris* Poivre 1981a, *Sagittalata jucunda* Poivre 1981a) that do conform to the genus *Sagittalata*. The pronotum them also differs between the genera: *Sagittalata tristis* and *Sagittalata tristella* have a smooth elongated pronotum with a thin metazona, again corresponding with *Cercomantispa* and not *Sagittalata hilaris*. In addition, the colour patterns of the prozona as well as the antennae also suggest these two species should be placed with *Cercomantispa* and not *Sagittalata*. Furthermore, the wing venation and genitalia suggest that *Perlamantispa* species conform to all the characteristics of *Sagittalata hilaris*. The only morphological difference is a subtle robustness of the pronotum. Some species of *Perlamantispa* seem to have a slightly more robust pronotum. *Perlamantispa* is consequently relegated as a synonym of *Sagittalata*. The difference between *Mantispa* and *Sagittalata* is weak as well, as explained in the systematic account above. Finding an autapomorphy proved to be difficult. The only consistent character was a continuous median longitudinal line on the anterior surface of the coxae of *Sagittalata* species. In addition to the coxal line, the presence of setae on the pronotum of *Mantispa* are lacking in the Afrotropical *Sagittalata*. However, two species did not conform to this character. The raptorial legs of *Perlamantispa* (= *Sagittalata*) *dorsalis* is completely black and therefore lacks the line. The second species, *Mantispa* (= *Sagittalata*) *nubila* lacks the line. Both species, however, lack setae on their pronotum. The rest of the *Sagittalata* species studied conforms to the characteristics. Distribution therefore plays an extremely important role in the delimitation of the genera. The type species of *Perlamantispa*, *Perlamantispa perla* however, is well known in Europe and *Mantispa styriaca* occurs in Morocco. C.-k. Yang (Yang and Peng 1998; Yang 1999) described three *Sagittalata* species from China. The descriptions are unfortunately in Chinese that could not be translated and the specimens were not studied. These species probably belong to *Mantispa* instead of *Sagittalata*. Except for these species the distribution patterns of the genera are quite clear with *Sagittalata* an Afrotropical genus and *Mantispa* a Palaearctic genus. Unfortunately it might be a genus that one will identify by eliminating other genera. Ongoing studies are in progress to find the relationship between *Sagittalata* and *Mantispa*.

#### Distribution.

##### 

Widespread in the Afrotropical Region. Also occur in the Palaearctic and Oriental Regions

#### Diagnosis.

An Afrotropical genus with four species currently known from the Palaearctic Region. Ectoprocts of males sligtly swollen (Fig. 4g), pseudopenis visible in dorsal view. Pterostigma elongated and dark red or black. Prothorax transversly rugulose; lacks setae (Fig. 3b). Fore coxae with continuous median line on anterior surface (Fig. 4b).

#### Description.

*Head*: Antennae moniliform. Flagellum dark, may end in two or three yellow flagellomeres. Anterior scape and pedicel either yellow or black; vertex flat, not visible in lateral view; frons and mouthparts vary in colour; eye margin yellow in dark species and black/dark brown in light species.

*Thorax*: Maculae inconspicuous, never pigmented in a different colour from the surrounding pronotum; pronotum lacks setae, transversely rugulose; prothorax longer than pterothorax.

*Wings* (Fig. 2c): Wings usually hyaline, may be partly or completely pigmented, pterostigma elongated and robust, always reddish or black; crossvein between radial cells 1 and 2 perpendicular to R; a single crossvein from third radial cell to anterior margin (C); *Hind wings*: Crossvein between Cu_a_ and Cu_p_+A_a_ attenuated, rarely absent; Cu_a_ with sharp angle to and from attenuated crossvein to form inverted triangle shape.

*Legs*: Raptorial legs differ in colour, coxal sulcus conspicuous, surrounding patterns never visible on sulcus; continuous line on anterior surface of fore coxae; fore tarsal claw reduced to a single claw lacking an arolium. Mid- and hind pretarsal claws pectinate (5–6 teeth); median tooth longer than surrounding teeth; pointed appearance; arolium present on mid and hind tarsi.

*Abdomen/Genitalia*: EEG present. Ectoprocts of male slightly swollen; slightly smaller than ectoprocts of members of *Afromantispa* and *Mantispa*; pseudopenis visible in dorsal view.

#### Discussion.

*Afromantispa*, *Mantispa* and *Sagittalata* seem to form a group with several similar aspects regarding their morphology. All three genera seem to have similar genitalic structures. In addition to the genitalia, the general wing venation is extremely similar with only the pterostigma of *Afromantispa* slightly different with a reddish, roundish and truncate appearance. In the hind wing, the inverted “V” shape made by the Cu_a_ when descending towards the attenuated or absent crossvein extending to Cu_p_+A_a_ and again ascending after the crossvein is prominent and easily identified in this group (Figs 2b, c). The median coxal line is not a strong autapomorphy since some of the *Mantispa* specimens studied had a discontinuous line on the anterior coxa, but the geographic distribution of the genera does support separate genera. A decision to keep the genera separate has consequently been made, thereby ensuring that relevant morphological information is not lost before a conclusive result is achieved. Of significant importance is the presence of the EEG that manifests in this group only.

### Genus Cercomantispa

Handschin

http://species-id.net/wiki/Cercomantispa

Cercomantispa Handschin, 1959: 224. Type species: *Mantispa mozambica* Westwood, 1852: 269 by original designation.

#### Remarks.

*Cercomantispa* is probably the most complex of all the Afrotropical genera. This is not only because of the sexual dimorphism and the general small size, but because of the confusion in the literature and physical state of the type specimen. Males are easily recognised by their elongated ectoprocts, but females do not have conspicuous genitalia and differ morphologically from the males in terms of colour and patterns. Females were therefore described as different species from the males and placed in several other genera. In addition to the confusion between the female *Cercomantispa* and *Sagittalata* there is a lack of clarity regarding the generic boundaries of *Cercomantispa*, *Necyla* and *Orientispa*. *Necyla* and *Cercomantispa* could be synonyms (Tjeder 1963). The name *Necyla* inexplicably disappeared from the literature. It is thought to be a genus comprising Oriental species with the type species *Necyla exigua* Navás being represented by a female holotype. The type species of *Necyla* could not be studied and photographs studied show a specimen in poor condition and pinned with closed wings. In addition to *Necyla* and *Cercomantispa*, male *Orientispa* Poivre 1984a also have elongated ectoprocts. The literature is not sufficiently unambiguous to synonymise these genera and for the purposes of this study, the genera *Necyla*, *Cercomantispa* and *Orientispa* will remain separate until further investigation can either confirm or reject the synonymy.

#### Distribution.

Widespread throughout the Afrotropical Region

#### Diagnosis.

The flagella of the antennae are very dark with the anterior surface of the scape and pedicel always yellow, even in the very dark species (Figs 3c, 4h). Pronotum smooth, lacks setae (Fig. 3c). The rectangular cell formed by the fusion of A_2_ and Cu_p_ in the hind wing is very diagnostic and no other mantispid genus has such a structure (Fig. 2d). All wing cells lacks pigment except for the pterostigma. The mid- and hind legs yellowish-brown to yellow covered in black setae. The males have elongated ectoprocts as well as an elongated pseudopenis, both longer than the 8^th^ tergite, and bent ventrally (Fig. 4f).

#### Description.

*Head*: Antennae long, moniliform; flagellomeres black; the apical three flagellomeres might be lighter in colour; anterior scape and pedicel always yellow, even in very dark species; vertex medially convex, clearly visible in lateral view; vertex bordered by conspicuous yellow eye margin; frons with longitudinal dark median line, not visible in very dark species *(e.g. C. tristis*); mandibles usually yellow or lighter than coloration of frons; black tipped with black inner margin

*Thorax*: Pronotum smooth, lacking setae; maculae conspicuous, not always pigmented; similar in length or slightly longer than pterothorax; in most species a dark median line forms two circular dorso-lateral yellow markings on prozona; prozona much wider than metazona, metazona narrow;

*Wings* (Fig. 2d): Wing venation comparatively simple; always lacks pigmented cells; pterostigma elongated, narrow; dark brown; a single crossvein from third radial cell to anterior margin (C); a single radial sector vein extending posteriorly from each radial cell 1, 2 and 3 respectively; four or five crossveins reaching posterior wing margin from M_p_ in hind wing; a rectangle shaped cell formed by the fusion of A_a_+Cu_p_ and Cu_a_.

*Legs*: Raptorial forelegs yellow; fore tarsal claw reduced to a single claw lacking an arolium; inner femoral surface dark in females; often only distal half dark in males (Fig. 4i), outer femur of both sexes with a narrow, brown latero-dorsal line; middle and hind legs yellow-brown to yellow covered in setae; most species with a narrow dark longitudinal line along femur and tibia; pretarsal claws pectinate; middle tooth projecting beyond the others giving the claw a sharp appearance.

*Abdomen/Genitalia: Male*: Ectoprocts elongated, longer than tergite 8; slightly swollen apically; apices bent downwards; pseudopenis elongated and bent ventrally; visible between ectoprocts in ventral and dorsal view; EEG absent

### Genus Rectinerva

Handschin

http://species-id.net/wiki/Rectinerva

Rectinerva Handschin 1959: 215. Type species: *Rectinerva braconidiformis*Handschin 1959: 221, by original designation.

#### Remarks.

*Rectinerva* is a monotypic genus. Only two female specimens have been collected, one being the holotype collected in 1933 (MRAC) the other collected in 1976 (MNHN) and described by Poivre (1985). The male remains undiscovered and sexual dimorphism therefore unknown.

#### Distribution.

Katanga (Democratic Republic of Congo) and Cameroon

#### Diagnosis.

Light red-brown. The antennal flagellae long slender and black, proximal half covered in prominent thick black setae (Fig. 4c). The anepimeron, anepisternum, katepimeron as well as katepisternum much lighter than the rest of the body, almost white (Fig. 4c). Three radial sector veins extending posteriad from radial cells 1–3. The wing colouration is unique among mantispids from the region (Fig. 2f).

#### Description.

*Head*: Head capsule light reddish-brown except for black tipped mandibles, vertex and pedicels. Scape light reddish-brown and pedicel black; flagellum long slender, black, proximal half covered in prominent thick black setae. Vertex medially raised in convex shape, visible in lateral view; raised vertex from antennal bases to posterior margin; black. Inner mandible margins lack black pigment. Eyes small; black to dark grey.

*Thorax*: Pronotum light reddish-brown; smooth; covered in light inconspicuous setae. Maculae inconspicuous; same colour as pronotum. Pterothorax uniform light red-brown; sutures inconspicuous and smooth; lacks deep clefts. Anepimeron, anepisternum, katepimeron as well as katepisternum lighter, almost white, conspicuous against the uniform light red-brown of the pteronota.

*Wings* (Fig. 2f): Both wings pigmented in banded formation with colours ranging from dark-brown to light red-brown. Pterostigma black and slightly concave in dorsal view. A single vein from radial cell 3 to the anterior wing margin (C). Radial cells broad, Radial cell 1 being the largest, radial cell 2 somewhat smaller and rectangular in shape with the radial cell 3 being the smallest. Lacks the hexagonal radial cell 4 found in other Afrotropical Mantispidae genera. Three radial sector veins extending in posterior direction from radial cells 1–3. *Hind wing*: Cu_a_ parallel with A_2_+Cu_p_. Cu_a_ - A_2_+Cu_p_ crossvein not attenuated and close to posterior margin.

*Legs*: Raptorial legs uniformly light reddish-brown; coxal sulcus same colour and inconspicuous; tibia-tarsal joint and fist tarsal segment black; fore tarsal claw reduced to a single claw lacking an arolium. Mid- and hind pretarsal claws pectinate (5–6 teeth); median tooth longer than surrounding teeth; pointed; arolium present on mid and hind tarsi. The rest of the mid leg light red-brown. Femur of hind leg light red-brown as well as the proximal third of the tibia, distal two-thirds and tarsal segments black; pre-tarsus light red-brown with some dark brown at pretarsal-claw bases.

*Genitalia*: At the time of this study the macerated female genitalia (prepared by Ragner Hall in 1983) were missing.

#### Discussion.

*Cercomantispa* and *Rectinerva* form a group because of synapomorphies. The male of *Rectinerva* is not yet known, so the genitalia cannot be used as a morphological character and sexual dimorphism cannot be excluded. However, the antennae of both genera are quite long compared to other Afrotropical taxa, and the flagellomeres are black with the anterior surface of the scape and pedicel yellow (Fig. 4h). Furthermore, the pronota of members of both genera are very similar in structure, smooth and narrow posterior to the maculae (Figs 3c, d). The rectangular shape of the cell formed by the fusion of A_a_+Cu_p_ and Cu_a_ is present in only *Cercomantispa*. However, the second cell between the Cu_p_+A_a_ and the posterior wing margin of *Rectinerva* is quite similar in shape but lacks the fusion between A_a_+Cu_p_ and Cu_a_. In addition to these, the comparatively simple wing venation and reduced number of radial sector cross veins in both genera seems to confirm the close relationship (Figs 2d, f).

### Genus Nampista

Navás

http://species-id.net/wiki/Nampista

Nampista Navás, 1914: 97. Type species *Nampista speciosa* Navás, 1914: 98 (= *Mantispa auriventris*Guérin-Méneville 1838: 202), by monotypy
Forciada Kozhanchikov, 1949: 355. Type species: *Forciada relicta* Kozhanchikov, 1949: 356 (= *Mantispa auriventris* Guérin-Méneville), by monotypy
Bucharispa Martynov, 1936: 437. Name unavailable, no type species was assigned to the genus.

#### Distribution.

Predominantly a Palaearctic genus In the Afrotropics the genus is found only in the countries bordering the Arabian Peninsula where it is represented by three species (Handschin 1960b, Ohl 2009).

#### Diagnosis.

The only Afrotropical genus close to *Nampista* is *Pseudoclimaciella*. It can easily be distinguished from *Pseudoclimaciella* by the following characteristics: Flagellomeres asymmetrically lamellate (Fig. 4d); deeply incised ventrally. Prothorax shorter than pterothorax (Fig. 3f). The basal half of the forewings always pigmented; the majority of the basal half of the hind wings clear (Fig. 2e).

#### Description.

Revised by Ohl (2009)

### Genus Pseudoclimaciella

Handschin

http://species-id.net/wiki/Pseudoclimaciella

Pseudoclimaciella Handschin, 1960a: 207. Type species: *Mantispa erichsonii*Guérin-Méneville 1844: 391 (as “*Pseudoclimaciella erichsoni* [sic] (Guérin-Méneville 1844)” on page 207, and as “*Climaciella erichsoni* [sic] Guérin-Méneville 1844” on page 210), by original designation. *Mantispa erichsonii* is a replacement name for *Mantispa grandis* Erichson, 1839: 164, which is a junior secondary homonym of *Mantispa grandis* Guérin-Méneville, 1831: 196.

#### Remarks.

*Pseudoclimaciella* can easily be recognised and confusion with other genera is unlikely. *Tuberonotha* Handschin 1961, a genus with a Palaearctic, Oriental and Australasian distribution is similar in appearance but always lacks an apical stain on the wings. Four Afrotropical species also lack an apical stain and were assigned to *Pseudoclimaciella* by either Handschin (*Pseudoclimaciella congensis* (Navás, 1936); *Pseudoclimaciella coronata* (Stitz, 1913)) or Poivre (*Pseudoclimaciella cachani* 1982b; *Pseudoclimaciella ivoiriensis* Poivre, 1982b) but all four have the characteristic elongated radial cells as well as a large number of crossveins originating from the radial cells. In addition to these, two crossveins originate from radial cell 3 and terminate at the anterior wing margin, all other Afrotropical mantispines have only one such vein. The members of *Pseudoclimaciella* are generally large mantispids. They may be mimics of certain Vespidae (Hymenoptera) species, an adaptation apparently not rare in Mantispidae. Colour patterns on the head as well as the pronotum fades with time and older museum specimens are frequently difficult to identify. Sexual dimorphism is unknown in *Pseudoclimaciella*.

#### Distribution.

The genus is probably confined to woodland and forests in the Eastern Tropical Corridor. All the locality data indicate a C-shaped distribution extending from the tropical areas in western Africa such as Sierra Leone through central Africa extending down into South Africa east of the plateau, and into Madagascar.

#### Diagnosis.

All members of *Pseudoclimaciella* are rusty reddish to brown. Basal cells of forewings always pigmented; basal cells of the hind wing always clear (Fig. 2a). Two or three veins originate from radial cell 3 and terminate at anterior wing margin (Fig. 2a); radial cells 1–3 elongated and narrow (Fig. 2a). Two yellow bands extend from pronotal maculae to ventral basal margin forming an inverted “V” shape on the dorsal side (Figs 3a, 4e). Hind tibia rusty reddish at joints and yellow in middle.

#### Description.

*Head*: Antennae moniliform; most flagellomeres dark in colour; twice as broad as long, one to three bright yellow flagellomeres apically; scape and pedicel rusty reddish. Vertex convex, not visible in lateral view; slightly raised posteriorly; vertex always yellow or rusty reddish; epistomal suture black except in *Pseudoclimaciella elisabethae* (Navás 1936); mandibles black tipped with black inner margin. Eye margins rusty reddish.

*Thorax*: Pronotal maculae conspicuous, from the pronotal maculae two yellow bands with black margins extend to ventral basal margin forming an inverted “V” shape on the dorsal side; prescutum with yellow margin forming another inverted “V” shape; prothorax longer than pterothorax, prozona relatively smooth, anterior margin of prothorax black, might be discontinuous medially; metazona with transversely rugulose, lacks setae. Postnota 2 and 3 often yellow as well as posterior abdominal margins providing a vespid wasp-like appearance.

*Wings* (Fig. 2a): Wing venation complex. Pterostigma elongated, narrow, rusty-reddish. Radial cells 1–3 elongated and narrow; of similar length; at least 8 radial sector veins extending in posterior direction from radial cells 1–3. Two veins from radial cell 3 extending towards anterior margin (C), (very few specimens with three such veins but only in one wing so some individual variation present). All species except for above mentioned 4 with apical pigmentation in both wings. *Hind wings*: Crossvein between Cu_a_ and Cu_p_+A_a_ prominent; Cu_a_ almost parallel with basal half of Cu_p_+A_a_; inverted triangle formed by Cu_a_ shallow.

*Legs*: Raptorial femur, tibia and tarsi uniformly red; lacks patterns on inner femoral surface; suture in fore coxa prominent and paler; single fore tarsal claw claw lacking an arolium. Mid- and hind pretarsal claws pectinate (5–6 teeth); teeth of similar size; spoon-shaped appearance; arolium present on mid- and hind-tarsi. Proximal joint of hind tibia dark rusty-red, distal joint lighter rusty red; proximad third of tibia same dark rusty red as joint, two distal thirds of tibia yellow.

*Abdomen/Genitalia*: Males with ectoprocts inconspicuous to slightly enlarged. Pseudopenis visible in lateral and dorsal view; continuous variation in both ectoproct size and pseudopenis size. EEG absent. No morphological significance regarding the female genitalia.

#### Discussion.

*Pseudoclimaciella* and *Nampista* are quite similar in many aspects. Species of both genera are generally quite large, reddish in appearance with wings often pigmented with a similar colour. From an Afrotropical perspective, they may form a group, but with other genera such as *Tuberonotha* from the Oriental Region that is in most aspects identical to *Pseudoclimaciella*, it is likely that *Nampista* is not the most closely related taxon to any of the Afrotropical genera.

### Key to the genera of the Afrotropical Mantispidae

**Table:** 

1a	Flagellomeres asymmetrical and lamellate (Fig. 4d)	*Nampista*
–	Flagellomeres symmetrical and moniliform (Fig. 4a, b, c, e)	2
2a	Crossvein between A_2_ and Cu_p_ in hind wing attenuated or absent (Fig. 2b, c, f)	3
–	A_2_ and Cu_p_ in hind wing fused (Fig. 2d) or crossvein between A_2_ and Cu_p_ in hind wing prominent (Fig. 2a)	4
3a	Prothorax granulated (Fig. 3e), white band present in distal third of antennae (Fig. 4a)	*Afromantispa*
–	Prothorax either smooth (Fig. 3c, d), rugulose (Fig. 3a, f) or covered in setae but lack granules (Fig. 3g) and white band in distal third of the antenna absent	7
4a	Prothorax smooth	5
–	Prothorax rugulose	*Pseudoclimaciella*
5a	Prothorax smooth and rectangle cell formed by fusion of A_a_+Cu_p_ and Cu_a_ in hind wing (Fig. 2d)	*Cercomantispa*
–	Prothorax smooth (Fig. 3d, 4c) but no fusion between A_2_ and Cu_p_ in hind wing (Fig. 2f)	*Rectinerva* (in part)
7a	In the hind wing Cu_p_ forms a sharp angle when bending towards A_2_ (Fig. 2c)	8
–	In the hind wing Cu_p_ do not form a sharp angle towards A_2_ (Fig. 2f)	*Rectinerva* (in part)
8a	Median line on anterior surface of fore coxae present (Fig. 4b), prothorax lacks setae (Fig. 3b), Afrotropical distribution	*Sagittalata*
–	Median line absent on anterior surface of fore coxae, setae present on prothorax (Fig. 3g), Palaearctic distribution	*Mantispa*

## The way forward

The taxonomic ultimate aim is to comprehensively revise the family. The present study will serve as a basis for future taxonomic research on the lower taxa of Afrotropical Mantispidae. The complexity of the Mantispidae has consequently been arranged into smaller monophyletic groups each with at least one autapomorphy. Future studies should therefore focus on revising each genus thereby avoiding an unnecessary increase in the already confusing list of invalid names in the region.

Genitalic structures may prove to be important to elucidate the species and should be thoroughly investigated. Given the diversity and complexity of the Afrotropical Mantispidae, molecular and behavioural studies may delimit what traditional morphological tools cannot resolve.

Fundamental knowledge regarding the basic biology of Mantispidae is sparse and requires attention. Studies that aim to investigate the oviposition preference of females, larval cues used for locating food sources and host specificity should be priority. This might allow for easier collection of specimens as well as raising other important questions regarding their evolution, behaviour and ecology.

## Supplementary Material

XML Treatment for Afromantispa

XML Treatment for Mantispa

XML Treatment for Sagittalata

XML Treatment for Cercomantispa

XML Treatment for Rectinerva

XML Treatment for Nampista

XML Treatment for Pseudoclimaciella
